# Myostatin inhibits glucose uptake via suppression of insulin‐dependent and ‐independent signaling pathways in myoblasts

**DOI:** 10.14814/phy2.13837

**Published:** 2018-09-03

**Authors:** Xin‐Hua Liu, William A. Bauman, Christopher P. Cardozo

**Affiliations:** ^1^ National Center for the Medical Consequences of Spinal Cord Injury James J. Peter VA Medical Center Bronx New York; ^2^ Department of Medicine Mount Sinai School of Medicine New York New York; ^3^ Department of Rehabilitation Medicine Mount Sinai School of Medicine New York New York

**Keywords:** Glut4, insulin resistance, muscle cells, myostatin

## Abstract

The glucose transporter 4 (Glut4) mediates insulin‐dependent glucose uptake. Glut4 expression levels are correlated with whole‐body glucose homeostasis. Insulin signaling is known to recruit Glut4 to the cell surface. Expression of Glut4 is subject to tissue‐specific hormonal and metabolic regulation. The molecular mechanisms regulating skeletal muscle Glut4 expression remain to be elucidated. Myostatin (Mstn) is reported to be involved in the regulation of energy metabolism. While elevated Mstn levels in muscle are associated with obesity and type‐2 diabetes in both human and mouse models, Mstn null mice exhibit immunity to dietary‐induced obesity and insulin resistance. The molecular mechanisms by which Mstn initiates the development of insulin resistance and disorders of glucose disposal are not well delineated. Here we investigated effects of Mstn on insulin action in C2C12 cells. Mstn significantly reduced basal and insulin‐induced IRS‐1 tyrosine (Tyr495) phosphorylation, and expression and activation of PI3K, associated with diminished AKT phosphorylation and elevated GSK3*β* phosphorylation at Ser9. In addition, Mstn inhibited Glut4 mRNA and protein expression, and reduced insulin‐induced Glut4 membrane translocation and glucose uptake. Conversely, SB431542, a Smad2/3 inhibitor, significantly increased cellular response to insulin. Mstn decreased AMP‐activated protein kinase (AMPK) activity accompanied by reduced Glut4 gene expression and glucose uptake, which were partially reversed by AICAR, an AMPK activator. These data suggest that Mstn inhibits Glut4 expression and insulin‐induced Glut4 integration into cytoplasmic membranes and glucose uptake and that these changes are mediated by direct insulin‐desensitizing effect and indirect suppression of AMPK activation.

## Introduction

Type 2 diabetes is associated with an impaired rate of insulin‐stimulated glucose disposal which has been attributed to insulin resistance in skeletal muscle (Marshall et al. 1993; Leguisamo et al. 2012). The glucose transporter 4 (Glut4) is the primary effector molecule for insulin‐mediated glucose disposal. Glut4 is expressed in adipose tissue, skeletal muscle and cardiac muscle cells; its expression levels are correlated with whole‐body insulin mediated glucose and lipid homeostasis (Liu et al. 1993; Ikemoto et al. 1995; Ranalletta et al. 2007; Atkinson et al. 2013). High levels of Glut4 increase insulin responsiveness. Mice that genetically overexpress Glut4, either globally or specifically in skeletal muscle or adipose tissue, display enhanced insulin responsiveness and peripheral glucose utilization (Treadway et al. 1994; Katz et al. 1996; Charron et al. 1999). Although skeletal muscle Glut4 expression is not compromised in diabetes or obesity (Anderson et al. 1993), skeletal muscle‐specific overexpression of Glut4 ameliorates insulin resistance (Leturque et al. 1996; Tsao et al. 1996). Increased Glut4 expression in skeletal muscle has also been reported to be associated with enhanced insulin sensitivity after exercise (Ikemoto et al. 1995; Ivy and Kuo 1998). Conversely, reduced Glut4 mRNA and protein expression have been reported in diabetic subjects (Berger et al. 1989; Sivitz et al. 1989). Thus Glut4 gene expression is a clinically relevant target for understanding the mechanisms of insulin resistance and diabetes.

Glut4 is directly regulated by the insulin‐induced intracellular signaling cascade. Upon insulin stimulation, insulin receptor substrate‐1 (IRS‐1), one of the major substrates of the insulin receptor, immediately undergoes tyrosine phosphorylation and binds to the Src homology 2 (SH2) domain of the p85 regulatory subunit of phosphatidylinositol 3‐kinase (PI3K) leading to activation of PI3K (Sun et al. 1991; Myers et al. 1994; Cantley 2002). Once active, PI3K promotes AKT (PKB) phosphorylation (Lizcano and Alessi 2002) and activation. AKT, in turn, enhances the phosphorylation and inactivation of glycogen synthase kinase 3*β* (GSK3*β*) leading to glucose storage as glycogen (Cantley 2002; Lizcano and Alessi 2002). Activation of the insulin‐signaling cascade also leads to a recruitment of Glut4 protein from an intracellular pool of vesicles to the cytoplasmic membrane, thus allowing glucose to enter the cell (Shepherd and Kahn 1999; Lizcano and Alessi 2002). Overexpression of constitutively activated PI3K and AKT (PKB) has been shown to stimulate the recruitment of Glut4 to the cell surface in the absence of insulin (Saltiel and Kahn 2001; Lizcano and Alessi 2002), whereas mice lacking a specific isoform of AKT (PKB) are diabetic and their insulin‐stimulated glucose uptake is impaired (Saltiel and Kahn 2001; Lizcano and Alessi 2002).

Although the regulation of Glut4 gene expression by insulin signaling has been widely studied, the data are not consistent. Insulin has been shown to have differential effects on Glut4 gene expression. Animals chronically treated with insulin show increased Glut4 mRNA in adipose tissue (Cusin et al. 1990; Postic et al. 1993), whereas chronic insulin treatment of 3T3L1 adipocytes has resulted in either no change or a marked reduction in Glut4 mRNA levels (Tordjman et al. 1989; Flores‐Riveros et al. 1993). On the other hand, metabolic regulation of Glut4 has been reported. Chronic fasting markedly reduces Glut4 mRNA levels in adipose tissue, while having either no effect or slightly increasing Glut4 mRNA in skeletal muscle (Charron and Kahn 1990). Moreover, incubation of 3T3L1 adipocytes in glucose‐free medium downregulates Glut4 mRNA about 10‐fold, whereas re‐addition of glucose to the starved adipocytes restores Glut4 mRNA levels (Tordjman et al. 1990; Charron et al. 1999). These data indicate that metabolic factors, rather than hormonal regulation, are dominant in the regulation of Glut4 gene expression.

More recently, several insulin‐independent pathways that regulate glucose metabolism and Glut4 gene expression have been reported. One of these is AMP‐activated protein kinase (AMPK). AMPK has been called the “fuel gauge” of mammalian cells and was first identified as a kinase for hydroxymethyl‐gutaryl‐CoA reductase (HMG‐CoA) and acetyl‐CoA carboxylase (ACC), key enzymes of steroid and fatty acid synthesis, respectively (Hardie et al. 1998). AMPK acts as a metabolic switch which is activated by an increase in the cellular AMP/ATP ratio. AMPK turns off ATP‐consuming processes, such as glycogen, fatty acid, and protein synthesis pathways, and activates alternative pathways for ATP regeneration including glucose transport, glycolysis, and fatty acid oxidation (Fisher et al. 2002; Jessen et al. 2003; Fujii et al. 2006). Activation of AMPK enhances sensitivity of skeletal muscle to insulin resulting in elevated insulin‐mediated glucose uptake (Merrill et al. 1997; Bergeron et al. 1999; Fisher et al. 2002; Fujii et al. 2006). In addition, AMPK activation has been demonstrated to directly upregulate Glut4 transcription and mediate exercise‐induced Glut4 content (McPherron et al. 1997; Jessen et al. 2003).

Myostatin (Mstn), a member of the transforming growth factor‐*β* (TGF‐*β*) superfamily, is a critical autocrine/paracrine inhibitor of skeletal muscle growth and development (McPherron et al. 1997). Beyond the confines of its traditional role, Mstn has recently been shown to play an important role in metabolism. The inhibitory effects of Mstn on insulin action were first postulated when Mstn null mice exhibited immunity to dietary‐induced obesity and insulin resistance (Gonzalez‐Cadavid and Bhasin 2004). More recently, Hittel et al. reported that injection of exogenous Mstn protein into mice leads to the development of insulin resistance (Hittel et al. 2009). Elevated levels of Mstn in muscle have been associated with obesity, type‐1 and type‐2 diabetes in both human and mouse models (Milan et al. 2004; Chen et al. 2009; Hittel et al. 2010). In addition, Mstn is thought to play a role in diabetic muscle atrophy as ob/ob diabetic mice have higher levels of myostatin expression (Allen et al. 2008). Genetic knockdown of Mstn‐suppressed body fat accumulation and protected from dietary‐induced insulin resistance (McPherron and Lee 2002; Zhao et al. 2005; Gao et al. 2009). The negative association of myostatin and metabolism is further supported by a transcriptomic array assay in which increased myostatin expression was observed in skeletal muscles of patients with type‐2 diabetes (Palsgaard et al. 2009). While a negative regulatory effect of Mstn on metabolism is widely reported, the literature contains some contradictory findings (Rodgers 2014; Rodgers et al. 2014). In vitro studies of myostatin effects on glucose metabolism showed increased glucose uptake using human placenta extracts (Mitchell et al. 2006) that contrasted to an inhibitory effect on glucose uptake seen in a placenta cell line (Antony et al. 2007). Furthermore, myostatin knockout mice show an increased AMPK activity in skeletal muscle, which could lead to increased insulin sensitivity (Zhang et al. 2011). In contrast, myostatin has been reported to increase AMPK activity in C2C12 myoblasts thereby improving glucose uptake (Chen et al. 2010). Moreover, the underlying molecular mechanism(s) that initiates the development of insulin resistance or disorders of glucose/fatty acid metabolism is not well delineated. Therefore, the effect and underlying mechanism(s) of Mstn action on insulin signaling and metabolism needs to be further elucidated.

## Experimental Procedures

### Cell line and cell culture

Mouse C2C12 cells were obtained from ATCC (Manassas, VA), and maintained in DMEM containing 10% FBS supplemented with 1% penicillin/streptomycin at 37°C. All experiments were performed with C2C12 cells that had been incubated for 48 h in DMEM containing 2% horse serum (HS) to initiate differentiation.

### Reagents

Recombinant myostatin protein was obtained from R&D System (Minneapolis, MN). Recombinant insulin protein was purchased from MBL International Co. (Woburn MA). Antibodies against phospho‐IRS‐1 (Tyr895), phospho‐PI3K, phospho‐GSK3*β* (Ser9), phosphor‐AKT (Ser473 & Thr308), phospho‐AMPK (Thr172), and endogenous IRS‐1, p85‐PI3K, p55‐PI3K, AKT, GSK3*β*, AMPK, and AICAR were purchased from Cell Signaling Technology (Beverly, MA). Anti‐Glut4 antibody was from Santa Cruz (Santa Cruz, CA). Antibodies against PGC1*α* and *β*‐tubulin were obtained from AbCam Inc. (Cambridge, MA). SB431542 was from Cayman Chem. Inc. (Ann Arbor, MI).

### Preparation of cell lysates and immunoblotting

C2C12 cells cultured under the desired conditions were lysed, as described previously (Liu et al. 2013). Briefly, cells were rinsed twice with ice‐cold PBS and scraped with 1.5 mL of PBS containing 4 mmol/L iodoacetate. After centrifugation, the pellets were resuspended in CHAPS extraction solution (10 mol/L CHAPS, 2 mol/L EDTA, pH 8.0, and 4 mol/L iodoacetate in PBS) with protease and phosphatase inhibitors. The samples were incubated for 30 min on ice and centrifuged at 15,000*g* for 10 min. The supernatants were collected and stored at −80°C. Protein from the membrane fraction was isolated using a commercial kit from Thermo Fisher Sci. (Rockford, IL), according to the manufacturer's instructions. For immunoblotting, cell lysates were electrophoresed on SDS‐polyacrylamide gels, electrophoretically transferred to a polyvinylidene difluoride membrane (Bio‐Rad, Herculus, CA), and incubated with targeting primary antibodies overnight at 4°C. Secondary horseradish peroxidase‐linked donkey anti‐mouse IgG (GE Healthcare, Buckinghamshire, UK) was then applied to the membranes and visualized by enhanced chemiluminescence (GE Healthcare).

### Quantitative real‐time (Rt) PCR

Rt‐PCR was performed as described previously (Liu et al. 2013) using a thermocycler (Model ViiA7, Thermo Fisher Sci.). For each sample, the determinations were performed in triplicate, and the means for the crossing points of triplicates were used in subsequent calculations. mRNA levels were expressed as fold‐change using the 2^−ΔΔCt^ method. Data were normalized relative to 18s RNA.

### Glucose uptake assay

Differentiating cells were treated with either vehicle, Mstn, or SB431542 for 3 days in glucose‐free culture medium. Cells were continuously incubated in serum‐free DMEM containing 0.1% BSA and treated with or without 100 nmol/L insulin for 30 min. Glucose uptake assays were then performed using an assay kit provided by Cayman Chemical. Inc., following the manufacturer's recommended procedures.

### Statistics

The data are expressed as mean values ± SEM. The significance of differences among means was tested by one way ANOVA with a Bonferoni test post hoc. Statistical calculations were performed using Prism 4.0 (Graph Pad Software, La Jolla, CA).

## Results

### Myostatin‐suppressed basal and insulin‐induced IRS‐1 tyrosine phosphorylation

**Figure 1:**
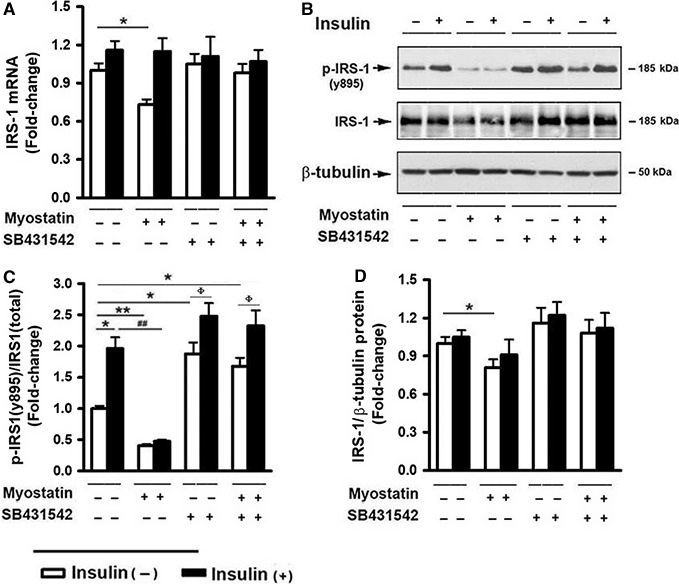
Myostatin‐suppressed basal and insulin‐induced IRS‐1 tyrosine phosphorylation. C2C12 cells were pretreated with either vehicle or myostatin (100 ng/mL), or SB431542 (10 *μ*mol/L) for 3 days, followed by treatment with insulin (100 nmol/L) for 30 min. (A) Total RNA was isolated and subjected to Rt‐PCR analysis. (B) Total cell lysates were prepared and subjected to Western blotting. (C) Blots in (B) were quantified by scanning densitometry and the ratio of phospho‐IRS‐1 (Tyr895) to endogenous IRS‐1 was calculated and is shown as fold‐change. (D) Blots in (B) were quantified by scanning densitometry and the ratio of endogenous IRS‐1 to *β*‐tubulin was calculated. Data shown in (B) are representative Western blot analysis; Data shown in (A, C and D) are mean values ± SEM from three separate determinations; *P < 0.05, and **P < 0.01 compared to vehicle only treated cells; ^#^ *P *< 0.05 and ^##^ *P *< 0.01 compared to insulin only treated cells; ^ϕ^ *P* < 0.05 compared to noninsulin‐treated controls.

The first intracellular step in insulin action after insulin binds the insulin receptor is tyrosine phosphorylation of IRS‐1 (Saltiel and Kahn 2001; Lizcano and Alessi 2002). We initially examined the effect of myostatin on IRS‐1 tyrosine phosphorylation in the presence or absence of insulin. As shown in Figure 1, In differentiating C2C12 myoblasts, insulin had no significant effect on IRS‐1 mRNA or protein expression but induced a notable increase in IRS‐1 tyrosine phosphorylation at Tyr895. In contrast, cells treated with Mstn showed a moderate, but significant downregulation in IRS‐1 mRNA and protein expression (Fig. 1A, B and D). Moreover, Mstn treatment markedly reduced basal and insulin‐induced IRS‐1 tyrosine phosphorylation (Tyr895) levels (Fig. 1B–C). To determine whether the inhibitory effect of myostatin is mediated by activation of Smad2/3 signaling, SB431542, a specific inhibitor of ALK5 that blocks phosphorylation of Smad2/3, was employed. When cells were treated with the inhibitor alone, there was a notable increase in basal IRS‐1 tyrosine phosphorylation (Tyr895) which was further elevated in the presence of insulin. When the cells were treated with both Mstn and SB431542, the inhibitory effect of Mstn was abolished. These data suggest that Mstn‐induced activation of Smad2/3 signaling may be involved in suppressing insulin action via preventing insulin‐induced IRS‐1 tyrosine phosphorylation. In addition, these results indicated that inhibitory effect of Mstn on IRS‐1 phosphorylation were Smad2/3 signaling dependent.

### Myostatin inhibited PI3K expression and activation

**Figure 2:**
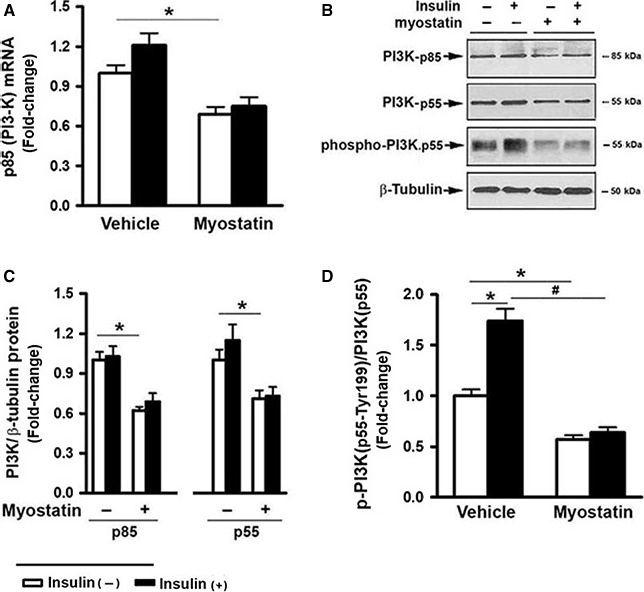
Myostatin inhibited PI3K expression and activation. C2C12 cells were pretreated with either vehicle or myostatin (100 ng/mL), or SB431542 (10 *μ*mol/L) for 3 days, followed by the addition of insulin (100 nmol/L) for 30 min. (A) Total RNA was isolated and subjected to Rt‐PCR analysis. (B) Total cell lysates were prepared and subjected to Western blotting. (C) Blots in (B) (upper panel) were quantified by scanning densitometry and normalized relative to *β*‐tubulin. (D) Blots in (B) (lower panel) were quantified by scanning densitometry and the ratio of phospho‐PI3K (p55‐Tyr199) to endogenous p55 was calculated and is shown as fold‐change. Data shown in (B) are representative Western blot analysis; Data shown in (A, C, and D) are mean values ± SEM from three separate experiments. *P < 0.05 and **P < 0.01 compared to vehicle only treated cells; ^#^ *P *< 0.05 compared to insulin only treated cells.

IRS‐1 tyrosine phosphorylation upon insulin binding to its receptor results in the recruitment of PI3K to the plasma membrane and leads to activation of PI3K, stimulating a significant increase in the concentration of PtdIns‐[3,4,5]‐P_3_, a key second messenger in the insulin signaling pathway (Cantley 2002; Lizcano and Alessi 2002). Thus, the effect of Mstn on the PI3K pathway was determined. While Mstn had no effect on the expression of p110, the catalytic subunit of PI3K, it inhibited mRNA expression of the regulatory subunit of PI3K, termed *Pik3r1* (Fig. 2A). This gene encodes 3 protein variants, that is, p85, p55*α* and p50*α*. Western blot analysis revealed while insulin had no effect, Mstn induced a moderate, but significant, inhibition in p85 and p55*α* protein expression compared to that seen in vehicle‐treated cells (Fig. 2B and C). However, insulin trigged a marked elevation in protein phosphorylation at Tyr199 of p55*α* (Fig. 2B); phosphorylation at Tyr458 of the p85 variant was not detected under the conditions of the experiment. In contrast, Mstn‐treated cells displayed decreased levels of both basal and insulin‐triggered p55*α* phosphorylation, suggesting a role for Mstn in suppressing endogenous PI3K activation and diminishing insulin action.

### Myostatin‐suppressed AKT phosphorylation and GSK3ß Ser9 phosphorylation

**Figure 3:**
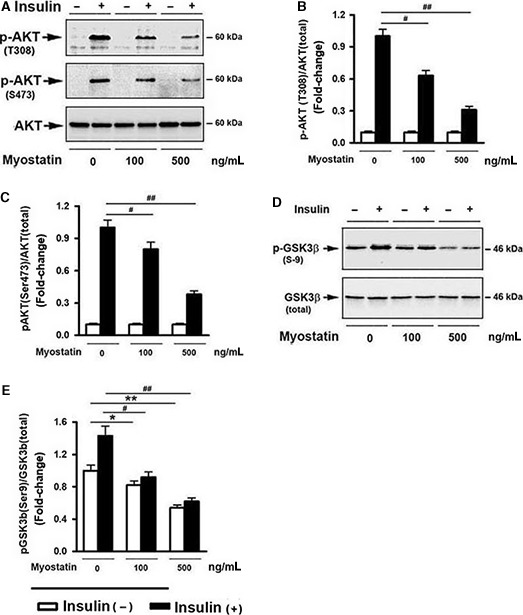
Myostatin‐suppressed AKT phosphorylation and glycogen synthesis. Cells were pretreated with either vehicle or various doses of myostatin, as indicated, for 3 days, followed by the addition of insulin (100 nmol/L) for 30 min. (A) Total cell lysates were prepared and subjected to Western blotting. (B) Blots in (A) (upper panel) were quantified by scanning densitometry and the ratio of phospho‐AKT (Thr308) to endogenous AKT was calculated and is shown as fold‐change. (C) Blots in (A) (middle panel) were quantified by scanning densitometry and the ratio of phospho‐AKT (Ser473) to endogenous AKT was calculated and is shown as fold‐change. (D) Western blot analysis on total cell lysates. (E) Blots in (D) were quantified by scanning densitometry and the ratio of phospho‐GSK3*β* (Ser9) to endogenous GSK3*β* was calculated and is shown as fold‐change. Data shown in (A and D) are representative Western blot analysis; Data shown in (C, D, E) are mean values ± SEM from three separate determinations. *P < 0.05, and **P < 0.01 compared of vehicle only treated cells; ^#^ *P *< 0.05, and ^##^ *P *< 0.01 compared to insulin only treated cells.

A key downstream effector of PI3K is AKT. AKT is activated by phospholipid binding and phosphorylation at Thr308 and Ser473 (Cantley 2002; Lizcano and Alessi 2002). The effect of Mstn on AKT phosphorylation was tested. Western blot analysis illustrated a dose‐dependent inhibition of AKT phosphorylation at both Ser473 and Thr308 sites in response to insulin stimulation after 3 day‐exposure to Mstn (Fig. 3A–C). The effect of Mstn on GSK3*β* phosphorylation at Ser9, which inhibits GSK3 activity, was determined. Insulin increased levels of GSK3*β* phosphorylated at Ser9 (Fig. 3D), and this change was diminished when the cells were pretreated with Mstn (Fig. 3E).

### Myostatin inhibited Glut4 expression and reduced glucose uptake by myoblasts

**Figure 4:**
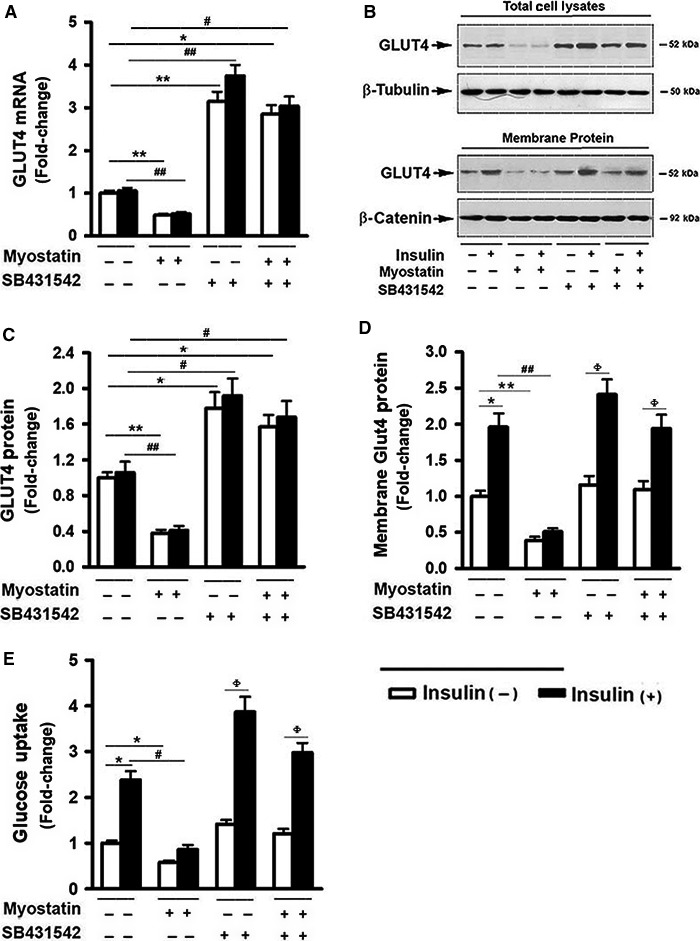
Myostatin inhibited Glut4 expression and reduced glucose uptake by myoblasts. Cells were pretreated with either vehicle or myostatin (100 ng/mL), or SB431542 (10 *μ*mol/L) for 3 days, followed by the addition of insulin (100 nmol/L) for 30 min. (A) Total RNA was isolated and subjected to Rt‐PCR analysis. (B) Total cell lysates and membrane protein were prepared and subjected to Western blotting. (C) Blots in B (upper panel) were quantified by scanning densitometry and normalized relative to *β*‐tubulin. (D) Blots in (B) (lower panel) were quantified by scanning densitometry and normalized relative to *β*‐catenin. Data shown in (B) are representative Western blot analysis; (E) Glucose uptake assay. Data shown in (A, C, D, and E) are mean values ± SEM from three separate experiments. *P < 0.05, and **P < 0.01 compared to vehicle only treated cells; ^#^ *P *< 0.05 and ^##^ *P *< 0.01 compared to insulin only treated cells; ^ϕ^ *P* < 0.05 compared to noninsulin‐treated controls.

Insulin‐activated PI3K and AKT pathways play critical role in glucose uptake by promoting recruitment of Glut4 from intracellular storage sites to the plasma membrane (Lizcano and Alessi 2002). The effect of Mstn on Glut4 expression, intracellular translocation, and glucose uptake rate were examined. Immunoblot analysis revealed a slight but not significant increase in Glut4 protein expression in the presence of insulin; however, insulin markedly upregulated of membrane Glut4 protein levels (Fig. 4B and D). In contrast, Mstn significantly inhibited Glut4 mRNA expression (Fig. 4A), reduced Glut4 protein levels (Fig. 4B and C), and abolished insulin‐induced elevation in the membrane fraction of Glut4 (Fig. 4B and D). Glut4 mRNA levels were significantly reduced after 24 h incubation with Mstn and plateaued at a new, lower level by 48 h after adding Mstn (Fig. S1). Glucose uptake was significantly reduced by 48 h after adding Mstn (Fig. S1). To determine whether this process is mediated by Smad2/3 signaling, the effects of SB431542 on Glut4 expression in the presence or absence of Mstn were tested. PCR analysis showed that SB431542 markedly upregulated Glut4 mRNA and protein expression (Fig. 4A–C). In addition, although SB431542 had no significant effect on basal membrane Glut4 expression, it significantly enhanced the insulin‐induced increase in Glut4 membrane protein levels (Fig. 4B and D). Glucose uptake assays revealed that treating cells with Mstn significantly inhibited basal and insulin‐induced glucose uptake (Fig. 4E). In contrast, SB431542 had no effect on basal glucose uptake, but caused a dramatic increase in glucose uptake in response to insulin stimulation (Fig. 4E). In addition, SB431542 blocked the inhibitory effects of Mstn on the expression of Glut4 mRNA and protein as well as glucose uptake (Fig. 4B–E). These data indicate an enhanced response of cells to insulin stimulation after inhibiting Smad2/3 signaling, suggesting an involvement of Smad2/3 signaling in regulating insulin sensitivity. In addition, the data imply that Mstn actions on Glut4 gene expression and glucose uptake are dependent on Smad2/3 signaling.

### Myostatin‐suppressed AMPK activation and downregulated Glut4 expression

**Figure 5:**
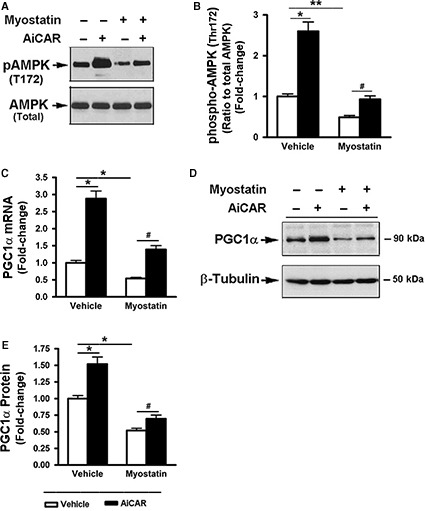
Myostatin‐suppressed AMPK activation and PGC1*α* expression. Cells were pretreated with either vehicle or myostatin (100 ng/mL) for 2 days, and further treated with AICAR (1 mol/L) for 18 h. (A) Total cell lysates were prepared and subjected to Western blotting. (B) Blots in (A) were quantified by scanning densitometry and the ratio of phospho‐AMPK (Thr172) to endogenous AMPK was calculated and is shown as percent of change. (C) Total RNA was isolated and subjected to Rt‐PCR analysis. (D) Total cell lysates were prepared and subjected to Western blotting. (E) Blots in (D) were quantified by scanning densitometry and normalized relative to *β*‐tubulin. Data shown in (A) and (D) are representatives of Western blot analysis; Data shown in (A, B, and E) are mean values ± SEM from three separate determinations. *P < 0.05, and **P < 0.01 compared to vehicle only treated cells; ^#^ *P *< 0.05 and ^##^ *P *< 0.01 compared to insulin only treated cells.

AMPK activity has been reported to regulate Glut4 transcription and glucose uptake (Fisher et al. 2002; Jessen et al. 2003; Fujii et al. 2006; McGee et al. 2008). In addition, Mstn‐deficient mice are reported to have elevated AMPK activity and reduced insulin resistance (Zhang et al. 2011). The effect of Mstn on AMPK activation and Glut4 expression was next examined. Cells treated with Mstn for 3 days showed a significant decrease in AMPK phosphorylation at Thr172 (Fig. 5A and B). In addition, Mstn suppressed the expression of PGC1*α* mRNA and protein, a direct down‐stream target of AMPK activation and a key regulator of mitochondrial biogenesis (Jager et al. 2007; Irrcher et al. 2008), supporting the conclusion that that signaling downstream of AMPK was increased as a result of AMPK T172 phosphorylation. These inhibitory effects of Mstn were partially reversed by the addition of AICAR, an activator of the AMPK signaling pathway (Fig. 5A–E).

### AICAR partially reversed Mstn‐induced inhibition in Glut4 expression and glucose uptake

**Figure 6:**
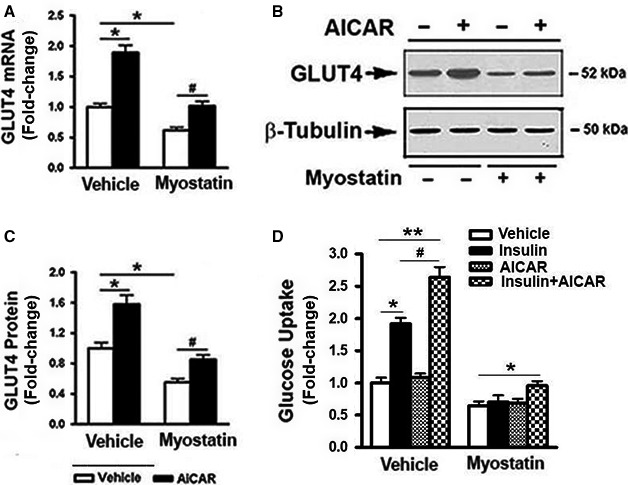
AICAR partially reversed myostatin‐induced inhibition of Glut4 expression and glucose uptake. C2C12 cells were pretreated with either vehicle or myostatin (100 ng/mL) for 2 days, followed by the addition of AICAR (1 mol/L) for 18 h. (A) Total RNA was isolated and subjected to Rt‐PCR analysis. (B) Total cell lysates were prepared and subjected to Western blotting. (C) Blots in (B) were quantified by scanning densitometry and normalized relative to *β*‐tubulin. (D) Myostatin‐pretreated cells were further treated with AICAR (1 mol/L, 18 h) and insulin (100 nmol/L, 30 min), alone or in combination, glucose uptake assay was then performed. Data shown in (B) are representative Western blot analysis; Data shown in (A, C, and D) are mean values ± SEM from three separate determinations. *P < 0.05, and **P < 0.01 compared to vehicle only treated cells; ^#^ *P *< 0.05 compared to insulin only treated cells.

To elucidate downstream consequences of AMPK inhibition by Mstn, the effects of AICAR on inhibition of Glut4 expression and glucose uptake by Mstn were evaluated in C2C12 myoblasts. Treatment of cells with Mstn significantly inhibited Glut4 mRNA and protein expression (Fig. 6A–C); this inhibitory action of Mstn was partially reversed by the addition of AICAR. The possibility that Mstn‐induced reduction in Glut4 expression was associated with a reduced rate of glucose uptake was tested. In the absence of Mstn, AICAR alone had no effect, but AICAR‐treated cells exhibited enhanced sensitivity to insulin stimulation. In the presence of Mstn, glucose uptake rate markedly decreased and was not increased by either insulin or AICAR alone. However, when the cells treated with a combination of AICAR and insulin, a modest but significant increase in glucose uptake was observed (Fig. 6D).

## Discussion

In young adults, skeletal muscle represents approximately 40% on body mass and is responsible for more than 60% of insulin‐mediated tissue glucose uptake. While many studies have shown that inhibition or knockouts of Mstn improve insulin sensitivity inferring an inhibitory effect of Mstn on insulin action, the cellular mechanisms by which Mstn achieves its biological actions are poorly understood. The studies reported herein used differentiating mouse C2C12 myoblasts, a biologically relevant cell culture model, to explore perturbations in insulin‐induced intracellular signaling responsible for a final effector of insulin action, specifically, expression of Glut4 and its translocation from cytosolic vesicles to the cytoplasmic membrane to augment cellular glucose uptake. An extensive analysis of Mstn‐induced changes in multiple potential mechanisms contributing to insulin resistance of muscle cells was conducted using a standard cell culture system thus permitting us to understand those changes that were concurrent. The data indicate that under the conditions of the experiment, Mstn causes multiple deleterious changes in signaling via IRS1/PI3K/AKT that include reduced levels of key signaling proteins and Glut4, and greatly attenuated insulin‐induced phosphorylation of proteins in the IRS1/PI3K/Akt signaling cascade. Moreover, Mstn blocked translocation of Glut4 to the cytoplasmic membrane and attenuated insulin‐induced glucose uptake into C2C12 cells. Finally, Mstn reduced AMPK phosphorylation and blunted AMPK activation by AICAR suggesting that Mstn may have other unappreciated effects on cellular metabolism. The possibility that these changes in AMPK phosphorylation reflect AMPK function is supported by data indicating that Mstn also blocked expression of one AMPK target gene, PGC1*α*. Because AMPK‐activated PGC1*α* activity plays an important role in regulating insulin‐induced glucose uptake and Glut4 expression (Merrill et al. 1997; Bergeron et al. 1999; Fisher et al. 2002; Fujii et al. 2006; Jager et al. 2007; Irrcher et al. 2008), the Mstn‐induced reduction in AMPK phosphorylation and PGC1*α* expression provides a plausible mechanism by which Mstn may reduce Glut4 gene expression. Future studies are required to formally test this prediction.

Myostatin signals by binding a heterodimeric cell surface receptor assembled from the Activin receptor IIB (ActRIIB) and ALK5. Binding of Mstn to ActRIIB activates the kinase domain of ALK5 leading to phosphorylation of Smad2 and Smad3 which are then translocated to the nucleus to regulate gene expression. SB431542, which inhibits ALK4/5/7 (Inman et al. 2002) and, thereby, prevents Smad2/3 phosphorylation, blocked inhibitory effects of Mstn on IRS1/PI3K/AKT signaling and on Glut4 expression, insulin‐induced translocation of Glut4 to cytoplasmic membranes and glucose uptake. This observation suggests that these inhibitory actions of Mstn require activation of Smad2/3. The most likely explanation for this apparent requirement for activated Smad2/3 involves reprogramming of gene expression through binding of Smad2/3/4 to cognate DNA sequences in target genes. Alternative explanations include tethering of Smads to other transcriptional regulators bound to chromatin, or interaction of activated Smads with other molecules involved in intracellular signaling. The possibility that activated ALK4/5/6 phosphorylate other proteins that govern insulin action, or that the inhibitor blocks kinases other than ALK4/5/6 must also be considered. The data reported here do not allow one to discriminate between these possibilities and this may be an interesting direction for future investigations.

Mstn also abrogated the insulin‐induced inhibitory phosphorylation of GSK3ß at Ser9. As noted above, inactivation of GSK3*β* by phosphorylation at Ser9 by activated AKT results in activation of glycogen synthesis. By preventing inactivation of GSK3ß, it would be expected that Mstn reduces activity of glycogen synthesis. Formal proof of this proposal will require further study.

Our findings are consistent with prior reports. Mstn has been reported to induce degradation of IRS1 (Bonala et al. 2014) and to inhibit PI3K/AKT/GSK3ß signaling (Morissette et al. 2006; Yang et al. 2007). Inhibition of Mstn augmented Glut4 expression and glucose uptake in vivo (Cleasby et al. 2014), and Mstn knockouts increase expression and phosphorylation of AMPK in skeletal muscle (Shan et al. 2013). Our data add to this literature by evaluating these multiple targets of Mstn action comprehensively in a relevant cell culture model of skeletal muscle. These data support a paradigm of Mstn action involving concurrent effects to reduce levels of key effectors in insulin receptor signaling, including both upstream (IRS1) and downstream (Glut4) targets of insulin action, coupled with greatly reduced ability of insulin receptor to activate intracellular signaling (Fig. S2). Such a model would be consistent with reprogramming of gene expression by activation of Smad2/3 as a consequence of myostatin action. The effects of Mstn on activation of AMPK and expression of PGC1*α* raise intriguing questions regarding the broader effects of Mstn on cellular energy metabolism.

Our study employed differentiating C2C12 mouse myoblasts as a biologically relevant model of insulin action on skeletal muscle. In our studies, Mstn was added 48 h after initiating differentiation, which is well after commitment to myogenic differentiation but prior to initiation of cell fusion, which begins at 3–4 days. We note, however, that Mstn has been reported to suppress proliferation and differentiation of myoblasts in culture through mechanisms that include reduced expression of the myogenic differentiation factor MyoD (Langley et al. 2002; McFarlane et al. 2011). Thus, a limitation of our studies is that they did not control for potential effects of any suppression of continued differentiation of C2C12 cells by Mstn on insulin signaling.

In conclusion, our findings indicate that in mouse C2C12 cells cultured under differentiating conditions that Mstn induces multiple, coordinated changes in intracellular signaling activated by insulin that, collectively, diminish insulin action and that these diverse effects are due to activation of Smad2/Smad3. Specifically, Mstn impairs signaling downstream of insulin receptor via IRS‐1/PI3K/Akt and insulin‐induced activation of AMPK and translocation of Glut4.

## Conflict of Interest

The authors have nothing to disclose.

## Supporting information




**Figure S1.** Effects of myostatin on Glut4 mRNA expression and glucose uptake in C2C12 cells.

**Figure S2.** Summary of myostatin‐induced insulin resistance.
