# Supplementary figures and images for: Myostatin inhibits glucose uptake via suppression of insulin‐dependent and ‐independent signaling pathways in myoblasts
Source: Physiol Rep. 2018 Sep 3;6(17):e13837. doi: 10.14814/phy2.13837 (PMC6121119; doi:10.14814/phy2.13837)

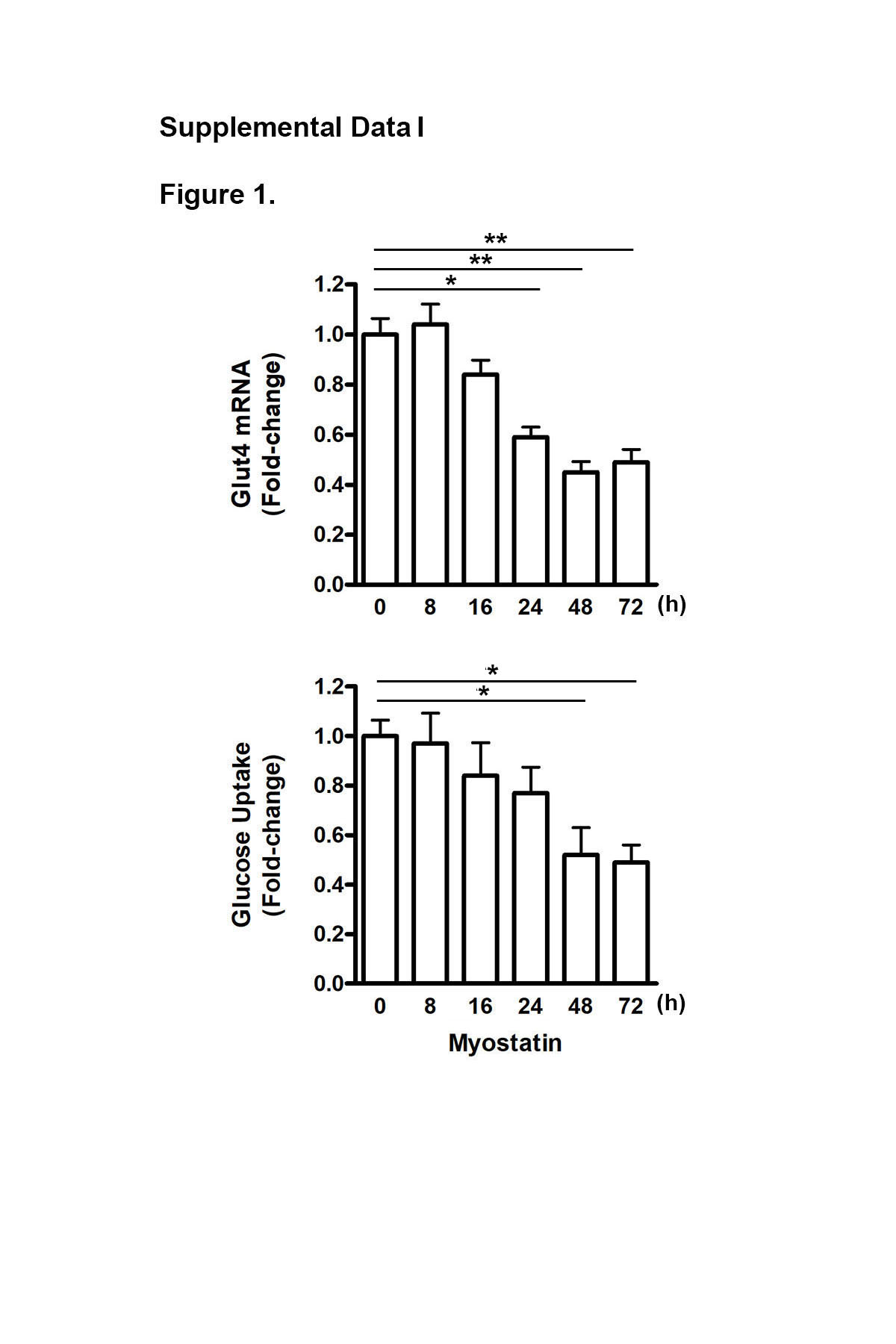

Supplement: Supplementary file 1 — Figure S1. Effects of myostatin on Glut4 mRNA expression and glucose uptake in C2C12 cells. [file PHY2-6-e13837-s001.jpg]

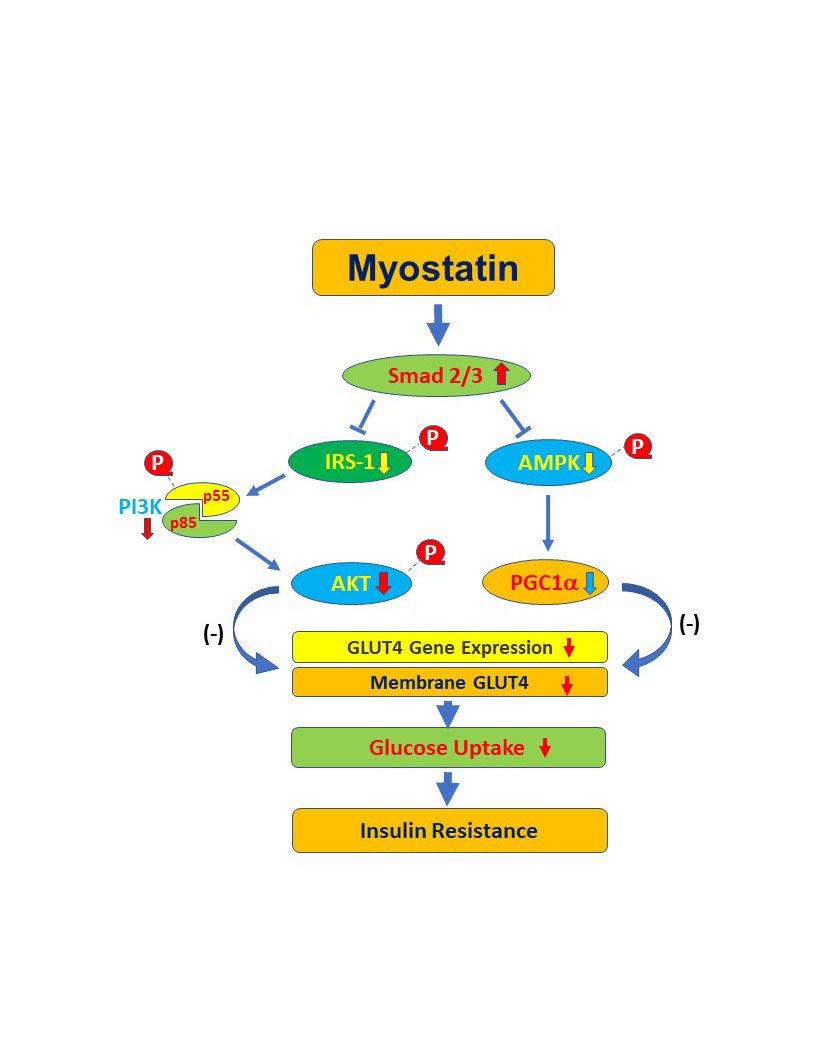

Supplement: Supplementary file 2 — Figure S2. Summary of myostatin‐induced insulin resistance. [file PHY2-6-e13837-s002.jpg]
